# Estimation of cell-free circulating *EGFR* mutation concentration predicts outcomes in NSCLC patients treated with EGFR-TKIs

**DOI:** 10.18632/oncotarget.14490

**Published:** 2017-01-04

**Authors:** Yan-juan Zhu, Hai-bo Zhang, Yi-hong Liu, Fu-li Zhang, Ya-zhen Zhu, Yong Li, Jian-ping Bai, Li-rong Liu, Yan-chun Qu, Xin Qu, Xian Chen, Yan Li, Guang-juan Zheng

**Affiliations:** ^1^ Oncology Department, The Second Affiliated Hospital of Guangzhou University of Chinese Medicine, Guangdong Provincial Hospital of Chinese Medicine, China; ^2^ Pathology Department, The Second Affiliated Hospital of Guangzhou University of Chinese Medicine, Guangdong Provincial Hospital of Chinese Medicine, China

**Keywords:** droplet digital polymerase chain reaction, epidermal growth factor receptor, non-small cell lung cancer, blood biopsy

## Abstract

Detection of circulating tumor DNA using droplet digital polymerase chain reaction (ddPCR) is a highly-sensitive, minimally invasive alternative to serial biopsies for assessment and management of cancer. We used ddPCR to assess the utility of measuring plasma concentrations of common epidermal growth factor receptor (*EGFR*) mutations (L858R, exon 19 deletion, and T790M) in 57 non-small cell lung cancer (NSCLC) patients treated with EGFR tyrosine kinase inhibitors (EGFR-TKIs). High baseline plasma *EGFR* mutation (*pEGFRmut*) concentrations were associated with shorter progression-free survival (8.43 months) than low baseline *pEGFRmut* (16.23 months; p = 0.0019). By contrast, there were no differences in tumor shrinkage or overall survival between groups. During EGFR-TKI treatment, *pEGFRmut* levels decreased to zero in 89.58% of patients. Twenty-five of the 27 patients who progressed had basal *pEGFRmut*, and 18 also had circulating T790M. All 20 patients with dramatic progression (according to a categorization system for EGFR-TKIs failure) had basal *pEGFRmut*, and 13 had T790M mutation at progression. These results support the use of ddPCR for analysis of plasma *EGFR* mutations for prediction of PFS and to monitor clinical responses to EGFR-TKIs in NSCLC patients.

## INTRODUCTION

Non-small cell lung cancer (NSCLC) is the most common type of lung cancer and the leading cause of cancer mortality worldwide [1]. Over the last decade, first-generation epidermal growth factor receptor-tyrosine kinase inhibitors (EGFR-TKIs) have been the first treatment choice for NSCLC patients who harbored TKI-sensitive *EGFR* mutations such as L858R (an amino acid substitution at codon 858 in exon 21), and ex19del (an in-frame deletion in exon19) [2–5]. In patients who are resistant to first-generation EGFR-TKIs but harbor the T790M mutation in exon 20 of the *EGFR* gene, third-generation EGFR-TKIs have shown efficacy [6, 7]. Therefore, assessing the presence of *EGFR* gene mutations is critical for treatment strategy decisions in NSCLC. Tumor tissue is still the recommended source for *EGFR* testing. However, tumor samples may not always be available or sufficient. Recently, circulating cell-free DNA (cfDNA) has attracted great attention because it can be easily obtained, and several technologies have been developed for its detection [8–10].

Droplet digital polymerase chain reaction (ddPCR) is a highly sensitive, quantitative analysis technology to detect gene mutations. Here, DNA is first emulsified with oil into thousands of droplets, each containing 0 or 1 DNA target molecules. Then, PCR amplification is performed in each fluorescently labeled droplet, so that droplets containing mutant or wild-type target DNA emit different color signals. Using a flow cytometer, the number of different color signals is read to calculate the concentration of target alleles [11]. We have focused on ddPCR for detecting *EGFR* mutations over several years. First, we demonstrated that ddPCR assays could achieve a detection sensitivity of 0.02% for mutant *EGFR* L858R, ex19del, and T790M, using tumor cells and normal human blood [12]. Second, we attempted to translate this technology to clinical diagnosis, and demonstrated that mutant plasma *EGFR* (*pEGFR*) concentration determined by ddPCR analysis could achieve a concordance of 86.73% with tumor *EGFR* (*tEGFR*) status [13]. Our previous data also revealed that high *pEGFR* mutation (*pEGFRmut*) concentrations correlated with extensive tumor burden [13]. Thus, albeit clinical validation is still needed, the quantitation of *pEGFRmut* may have great prognostic value for NSCLC. With the goal of developing a robust biomarker assay to predict prognosis in patients treated with EGFR-TKIs, we present here a ddPCR quantitative analysis of *EGFR* mutations (L858R, ex19del, and T790M) using cfDNA isolated from the plasma of 57 NSCLC patients.

## RESULTS

### Patient characteristics

Table 1 shows baseline data for the 57 patients treated with EGFR-TKIs. No significant differences were observed in demographic or clinical characteristics between patients with different types of *tEGFR* mutation *(tEGFRmut)*. EGFR-TKI therapies were similar for patients with either L858R or ex19del mutations. No significant differences were found in qualitative *pEGFR* status or quantitative *pEGFRmut* concentrations between patients with tumor-positive L858R and ex19del mutations.

**Table 1:** Demographic, clinical and therapeutic information of the 57 patients treated with EGFR-TKIs

Characteristic	*EGFR* Status in Tumor Tissue			p
	Total	L858R	ex19del	
Gender, male/female (n=57)	23/34	16/18	7/16	0.289^a^
Age(year), mean±SE (n=57)	65.05±1.41	66.26±1.69	63.26±2.46	0.2456^c^
Smoking history, yes/no (n=57)	15/42	11/23	4/19	0.375^b^
ECOG performance status, 0-1/2-4 (n=57)	49/8	29/5	20/3	0.605^b^
Histology, adeno-/squamous cell carcinoma (n=57)	57/0	34/0	23/0	—
Clinical stage, III/IV (n=57)	7/50	3/31	4/19	0.177^b^
Bone metastasis, yes/no (n=49)	31/18	20/11	11/7	0.907^a^
Brain metastasis, yes/no (n=52)	21/31	10/22	11/9	0.083^a^
Liver metastasis, yes/no (n=57)	11/46	6/28	5/18	0.380^a^
Contralateral lung metastasis, yes/no (n=57)	22/35	15/19	7/16	0.154^a^
EGFR-TKIs, gefitinib / erlotinib/other (n=57)	36/19/2	20/13/1	16/6/1	0.627^b^
*EGFR* status in plasma, positive/negative (n=57)	46/11	27/7	19/4	0.522^b^
EGFR mutation concentration in plasma, median (25% ~75% percentile) (n=57)	189.6 (6.2~477.4)	222.1 (12.6~477.4)	75 (3.8~607.1)	0.7689^d^

^a^ χ^2^ test;

^b^ Fisher's exact test;

^c^ t test;

^d^ Rank sum test.

### Association with progression-free survival, overall survival, and tumor response

**Figure 1:**
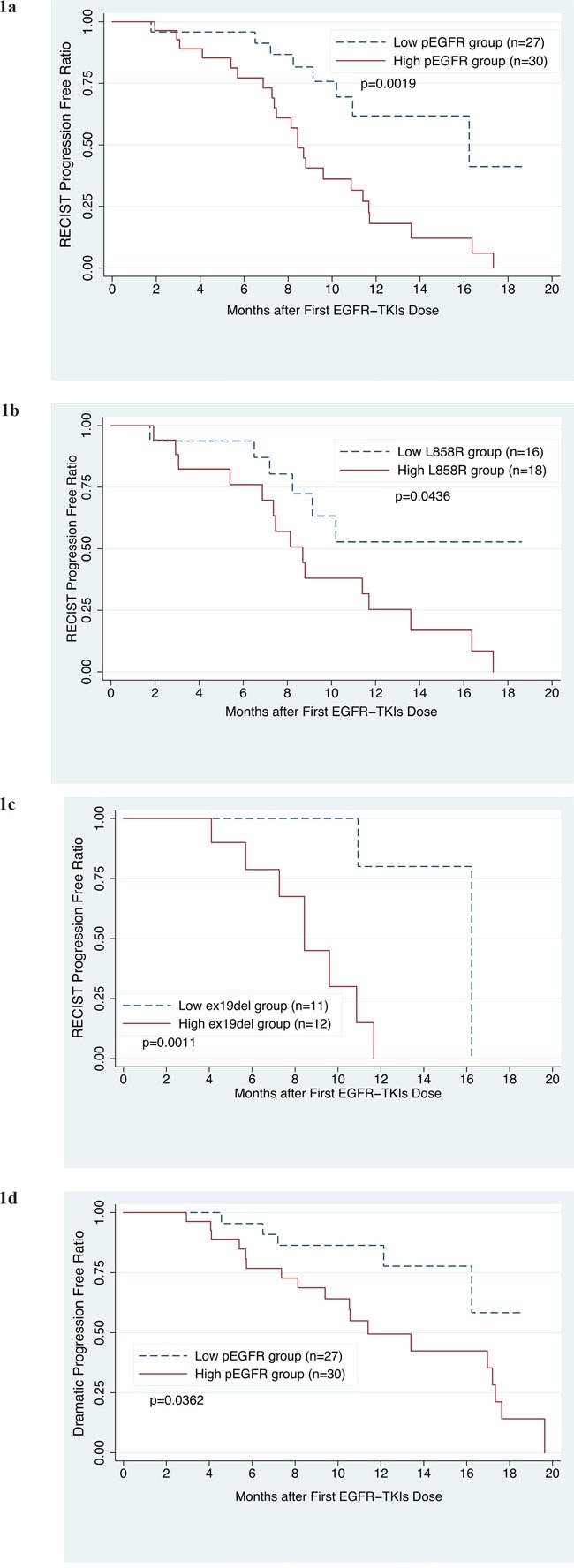
Progression-free survival curves for the 57 patients treated with EGFR-TKIs **1a**. PFS of patients with high or low basal plasma *EGFR mutation (pEGFRmut)* levels. **1b**. PFS of patients with high or low basal plasma L858R mutation levels. **1c**. PFS of patients with high or low basal plasma ex19del mutation levels. **1d**. PFS of patients with dramatic disease progression, grouped according to high or low basal *pEGFR*mut levels. High *pEGFRmut* was defined as having mutant *pEGFR* concentrations higher than the median, that is, a L858R concentration ≥200copies/ml or an ex19del concentration ≥75copies/ml.

Median follow-up for the 57 patients was 12.27 months (range, 0.5-22.23 months). By the end of follow-up, 31 patients (54.39%) had progressed, as determined by imaging and the Response Evaluation Criteria in Solid Tumors (RECIST) v1.1 guidelines. Median progression-free survival (PFS) for high and low baseline *pEGFRmut* concentration was 8.43 and 16.23 months (p = 0.0019, Figure 1a), respectively. Patients with a *pEGFRmut* concentration greater than the median—that is, ≥200copies/ml for L858R or ≥75copies/ml for ex19del—were included in the high *pEGFRmut* group. The same results were found when we analyzed L858R and ex19del separately. Median PFS was 8.7 months for high L858R patients, whereas median PFS for low L858R patients was not reached (p = 0.0436). For high and low ex19del patients, median PFS was 8.43 and 16.23 months (p = 0.0011), respectively (Figure 1b, 1c). In the multivariate Cox regression model, *pEGFRmut* concentration, smoking history, and brain metastasis were independently associated with PFS, when adjusted for tumor burden, sex, ECOG performance status, stage, and contralateral lung-, liver-, bone-, and adrenal metastases [hazard ratio (HR) = 3.96; 95 % confidence interval (CI), 1.10-14.22; p = 0.035; Table 2 ].

**Table 2:** Cox regression for RECIST progression-free survival, n=48

Variables	HR	SE	95% CI	p
Group of *pEGFR* (high=1, low=0) ^a^	3.96	2.58	1.10-14.22	0.035
Smoker (yes=1, no=0)	4.61	3.57	1.01-21.04	0.049
Brain metastasis (yes=1, no=0)	3.28	1.87	1.07-10.02	0.037

Justified by tumor burden, gender, ECOG performance status, stage, contralateral lung metastasis, live metastasis, bone metastasis, and adrenal metastasis;

HR, hazard ratio; SE, standard error; CI, confidence interval;

^a^ High group, patients with mutant *pEGFR* concentration more than the median (L858R≥200copies/ml or ex19del ≥75copies/ml).

In clinical practice, most patients continue taking the originally prescribed EGFR-TKIs, even after progression is detected by imaging. Therefore, to guide subsequent management, Jin-ji Yang et al. proposed a series of clinical modes of EGFR-TKIs failure, classified as *dramatic* progression, *gradual* progression, and *local* progression. In their study, a switch to chemotherapeutic regimens showed a modest survival benefit only when progression was deemed dramatic. Dramatic progression was defined as: 1) disease control lasting ≥3 months with EGFR-TKI; 2) deterioration of any pre-existing or new symptoms, including cough, hemoptysis, chest pain, fever, dyspnea and metastatic lesion-related symptoms; and 3) rapid progression of multiple measurable lesions compared with the previous assessment, or at least 2 of the following progressive involvement of non-measurable lesions, including progression of pre-existing lesions; progression due to new lesions in the thoracic cavity; new lesions beyond the thoracic cavity; or new malignant effusion [14]. By the end of follow-up, according to Jin-ji Yang's modes, 40.35% (23/57) of the patients in our study showed dramatic progression. Among these, median PFS was 11.4 months for those with high baseline *pEGFRmut* concentration, whereas PFS for the low-*pEGFRmut* group was not achieved (p = 0.0362, Figure 1d). No significant differences were found when we analyzed L858R and ex19del separately.

**Figure 2:**
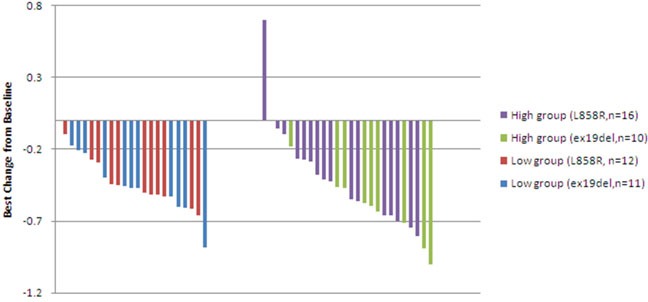
Best shrinkage from baseline tumor burden during EGFR-TKI treatment in patients with high or low basal pEGFRmut levels High *pEGFRmut* was defined as having *pEGFRmut* concentrations ≥200copies/ml for L858R and ≥75copies/ml for ex19del.

By the end of follow up, 29.82% (17/57) of the patients had died. Median overall survival (OS) for high and low *pEGFRmut* groups was 16.1 months and 17.33 months (p = 0.1763), respectively. Tumor response was assessed in 49 patients. According to RECIST v1.1, in the low-pEGFRmut group there were 19 patients with partial response (PR), three with stable disease (SD), and one with progressive disease (PD), while in the high-pEGFRmut group 22 patients had PR, two had SD, and two had PD (p = 0.862). No significant difference in shrinkage of measurable lesions was observed between the high and low *pEGFRmut* groups, with mean best shrinkage from baseline of 44.22 ± 6.57% in the high *pEGFRmut* group and 40.16 ± 4.58% in the low *pEGFRmut* group (p = 0.6142; Figure 2).

### Treatment monitoring values

Forty-eight patients were followed up with *pEGFRmut* testing. ddPCR flow cytometry plots for two typical patients are shown in Supplementary Figures 1 and 2. During EGFR-TKI therapies, mutant *pEGFR* concentrations decreased to zero in 43 patients (89.58%). The other five patients, in whom *pEGFRmut* remained detectable during treatment, had significantly poorer PFS and included two without response and three who progressed in 4, 5, and 8 months. The concordance between *pEGFRmut* testing and imaging examination at follow-up during EGFR-TKI therapy is shown in Table 3. Of the 27 patients who progressed, 13 had detectable levels of baseline *pEGFRmut* at least 2 months before progression, nine had positive *pEGFRmut* at progression, and three were positive 2 and 4 months after progression. In two patients with gradual progression, no mutation was detected at progression or after 2 and 4 months. All the 20 patients with dramatic progression that were followed up had previous detectable *pEGFRmut*, and fifteen were *pEGFRmut* positive at least 2 months before dramatic progression. The other five were positive at dramatic progression (Table 3).

**Table 3:** Concordance at follow-up between *pEGFR* testing and imaging findings during EGFR-TKI therapy

		pEGFR(L858R or ex19del)		Concordance Rate(95% CI)	Sensitivity(95% CI)	Specificity(95% CI)	PPV(95% CI)	NPV(95% CI)
		(+)	(-)					
RECIST progression	Yes (27)	25	2	93.75%	92.59%	95.23%	96.15%	90.91%
	No (21)	1	20	(86.90-100%)	(82.71-100%)	(86.12-100%)	(88.76-100%)	(78.90-100%)
Dramatic progression	Yes (20)	20	0	87.5%	100%	78.58%	76.92%	100%
	No (28)	6	22	(78.14-96.86%)		(63.38-93.78%)	(60.72-93.12%)	

CI, confidence interval; PPV, positive predictive value; NPV, negative predictive value.

Twenty patients had detectable *pEGFR* T790M mutation during the follow-up period. Among these, 18 suffered progression and showed detectable levels of L858R or ex19del at follow-up. Among these, T790M was detected earlier than *pEGFRmut* in three patients, concurrently with *pEGFRmut* in twelve patients, and after *pEGFRmut* in the remaining three patients. Five of these 18 patients underwent re-biopsy at progression and were found to have *tEGFR* T790M. Notably, we did not detect the T790M mutation in plasma from three patients with dramatic progression who harbored tumor tissue T790M mutation at progression.

## DISCUSSION

Routine testing of *EGFR* gene mutations is a fundamental step before selecting a treatment strategy for NSCLC patients. For screening these and other cancer-related mutations, liquid biopsy has attracted much attention due to its minimally invasive nature. Our previous data [13], as well as results reported in other studies [15, 16], demonstrated that ddPCR is a useful clinical diagnostic method for *pEGFR* detection, with a concordance of 80-90% with *tEGFR* status. Moreover, in addition to its high sensitivity, as a quantitative technology ddPCR has the advantage of providing concentration profiles of target alleles. Although several studies addressed this issue, the clinical significance of estimating *pEGFRmut* concentrations in NSCLC has not been unequivocally established. In this study, we found that higher baseline *pEGFRmut* levels were correlated with poorer PFS. This result is in line with results from other ddPCR studies showing an inverse correlation between circulating tumor DNA (ctDNA) and/or tumor-associated transcripts and clinical outcome. For instance, in a study of melanoma patients who received *BRAF* inhibitors, low concentration of plasma *BRAF*^V600E^ was significantly associated with longer OS and PFS [17]. In a breast cancer study, high serum miR-10b-5p levels were found to be associated with clinical biological markers of poor prognosis [18]. Subgroup analysis in the EURTAC trial of NSCLC patients treated with erlotinib also supported our findings. Using the TaqMan assay, a qualitative method, to detect *EGFR* mutations in plasma, it showed that median OS was significantly shorter in patients with both tumor and plasma L858R mutation (13.7 months) than in tumor-only L858R-positive patients (27.7 months) 19]. However, in another study in which the consistency between qualitatively detected *pEGFR* and *tEGFR* was compared in NSCLC patients, the authors also reported the objective response rate in 106 observed patients. Objective response rate to the EGFR-TKI gefitinib was 76.9% (95% CI, 65.4-85.5%) in patients with both *pEGFR* and *tEGFR* mutations, and 59.5% (95% CI, 43.5-73.7%) in *tEGFR*-positive but *pEGFR*-negative patients [20]. Since tumor lysis has been postulated to be the main source of ctDNA [21, 22], a possible explanation for these seemingly discordant results is that *pEGFRmut* concentration reflects *EGFR* mutation abundance in tumor tissues, which in turn might entail higher sensitivity to EGFR-TKIs. If this is the case, it is possible that higher *pEGFRmut* levels might, at least in some patients, predict better response to EGFR-TKIs [20].

However, as discussed below, additional factors are likely to be at play. In agreement with earlier studies [21, 22], including our own [13], we showed that *pEGFRmut* concentration was indeed correlated with tumor burden. Using ddPCR, a recent study found that the overall incidence of TKI-naïve (pretreatment) T790M mutation in tumor tissues was 79.9%, with a frequency ranging from 0.009% to 26.9%. T790M was detected more frequently in larger tumors [24]. Since the point mutation T790M in the EGFR kinase domain is the major mechanism of resistance to EGFR-TKI therapies, it is conceivable that patients with higher *pEGFRmut* concentrations progress at a faster rate because of extensive tumor burden and potentially higher frequency of pretreatment T790M mutation.

In our study, a significant difference in PFS, but not in OS, was revealed in patients with low, compared with high, *pEGFRmut* plasma levels. One possible reason for the lack of difference in OS between groups may be that 16 progressed patients received second-line therapies, including 8 that were treated with AZD9291. The efficacy of second-line therapies would reduce the difference in OS between the two groups. In contrast, in the melanoma and NSCLC (EURTAC) studies referred above, patients who progressed after treatment with, respectively, *BRAF*^V600E^ inhibitors or erlotinib, did not receive effective second-line therapies. In addition, the short follow-up period may have precluded finding significant differences in OS in our patients.

Several studies have assessed the value of liquid biopsy in monitoring efficacy during treatments. In a small sample study of *pEGFRmut* in NSCLC, *pEGFRmut* concentration decreased to zero in most patients [16]. In the aforementioned melanoma study, *cfBRAF*^V600E^ concentrations decreased significantly in the first month of *BRAF* inhibitor therapy and at the moment of best response [17]. Another NSCLC study found a significantly shorter median PFS of 6.3 months in patients with detectable *pEGFR*, compared with 10.1 months for those with undetectable *pEGFR* after two-month EGFR-TKI treatment [15]. Similarly, our study showed that *pEGFRmut* concentrations were reduced to zero in 89.58% of patients, and these had a significantly better PFS than those who retained detectable levels of *pEGFRmut*. Also, although basal *pEGFRmut* was not detected in some patients who progressed, all 20 patients who showed dramatic progression had detectable pretreatment levels of *pEGFRmut*. We suggest that in clinical practice, dramatic progression criteria assessment is much more important than imaging criteria for progression, for in many circumstances, a change in treatment strategies is advisable only when progression is dramatic after failure of EGFR-TKIs [14]. Considering the risk of radiation exposure and the economic cost of imaging examinations, we propose that serial plasma genotyping by ddPCR should be the first screening test to guide treatment decisions, and imaging should be used only after *pEGFRmut* status is determined. Further studies are needed to determine the feasibility of this proposed monitoring model.

In conclusion, we found that pretreatment *pEGFRmut* concentration determined by ddPCR was positively correlated with poorer PFS after EGFR-TKI therapy. This assay also displayed a satisfactory monitoring value during EGFR-TKI treatment.

## MATERIALS AND METHODS

### Patients and treatments

**Figure 3:**
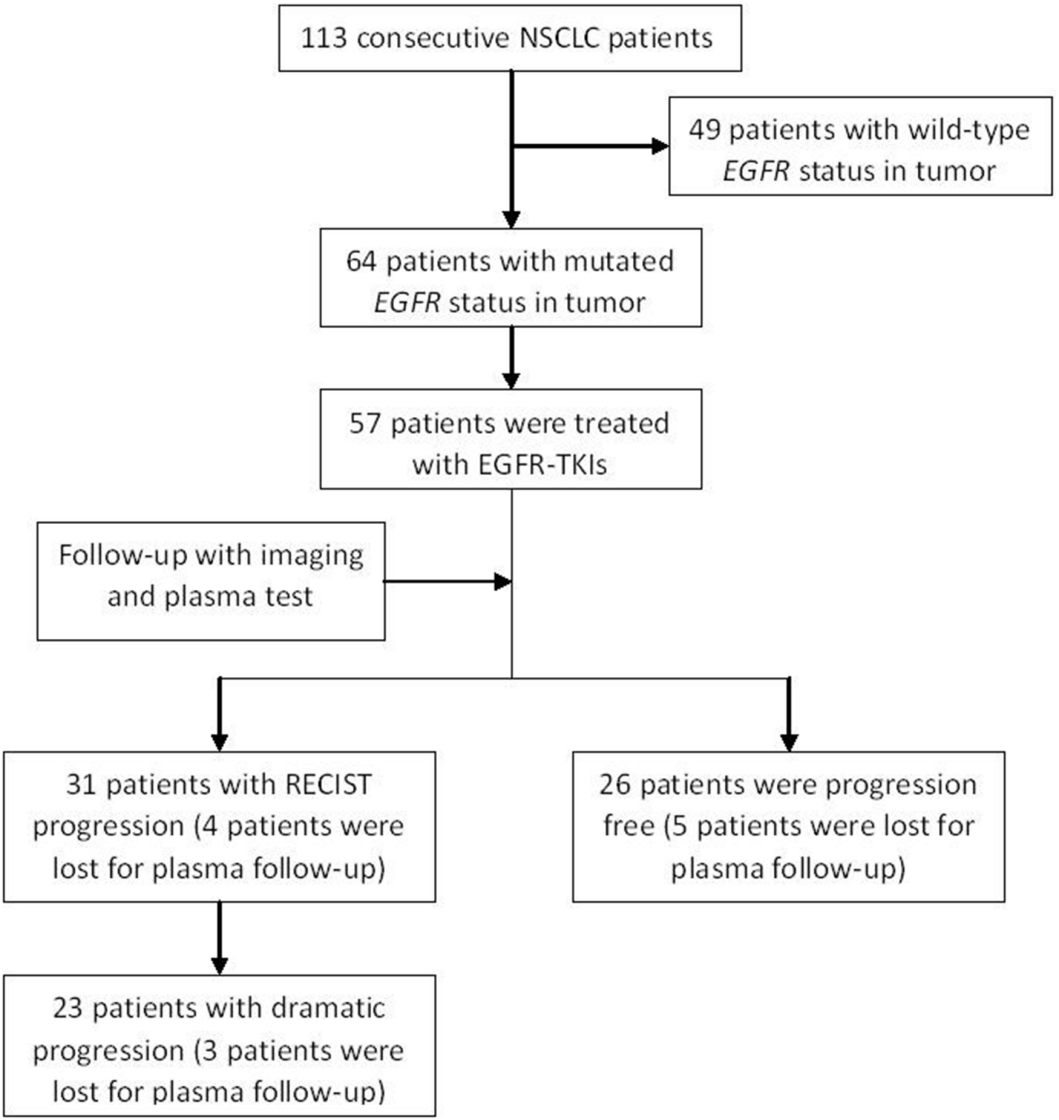
Study flow diagram

In our previous observational study, we consecutively enrolled 113 previously untreated NSCLC patients from October 2014 to May 2016, including 64 patients who harbored mutated *tEGFR*. Patients who had other uncontrolled malignant tumors, uncontrolled infection or *Mycobacterium tuberculosis*, or severe mental disease were excluded. Physicians and patients made treatment decisions together, and 57 stage III/IV patients with TKI-sensitive *tEGFRmut* received EGFR-TKIs as first-line therapy. These 57 patients were enrolled in this study, and undergo a follow-up imaging examination every 2 months, if possible, with a maximum interval of 3 months (Figure 3). All patients provided written informed consent for this study and for the *EGFR* gene test. This study was approved by the Ethics Committee of Guangdong Provincial Hospital of Chinese Medicine.

### Plasma collection and ddPCR workflow

For each patient, baseline plasma was collected before first-line therapy. Additional follow-up plasma for patients who received EGFR-TKIs was collected every 2 months, with a maximum interval of 3 months, at the same time of imaging. 6-10 ml of whole venous blood were collected into EDTA-containing vacutainers, stored at 4°C, and centrifuged (10 min, 1800g at 4°C) within 6 hours of collection. Plasma was frozen at -80°C until use. The ddPCR workflow (as detailed in Supplementary Methods) was conducted at WuXi AppTec Medical Testing Institute (Shanghai) Co., Ltd. Scientists conducting the ddPCR were blinded to the tissue results.

### Statistical analysis

PFS of patients treated with EGFR-TKIs was the primary endpoint for assessing the relationship between *pEGFRmut* concentration and prognosis. PFS was defined as the time between the date of first EGFR-TKI dose to the date of progression determined by imaging, according to the RECIST v1.1 guidelines. Patients were followed up until August 2016. Those who were progression-free at the end of the study or lost to follow-up were censored. Secondary endpoints included OS, tumor response rate, and best shrinkage of measurable lesions. OS was defined as the time from the date of first EGFR-TKI dose to the date of death. Patients who survived until the end of the study or who were lost at follow-up were censored. Best tumor response, such as complete response, PR, SD, and PD, was assessed according to RECIST v1.1. Measurable lesions were measured as the sum of the longest diameters according to RECIST v1.1, and the best shrinkage was the maximum reduction percentage of measurable lesions during treatment compared to baseline. Differences in demographic and clinical characteristics between the two groups were evaluated using the t test, rank sum test, χ^2^ test, or Fisher's exact test, with an alpha < 0.05. PFS and OS curves were calculated using the Kaplan-Meier method and compared with the log-rank method, with an alpha < 0.05. The multivariate Cox model to estimate hazard ratios (HR) and 95% CI was also used for comparison of PFS. Data were documented using EpiData software (version 3.1, The EpiData Association, Odense, Denmark) and analyzed using Stata software (version 11.0, StataCorp LP, College Station, TX, USA).

## SUPPLEMENTARY DATA



## Abbreviations

EGFR: Epidermal Growth Factor Receptor
ddPCR: Droplet Digital Polymerase Chain Reaction
NSCLC: Non-small Cell Lung Cancer
EGFR-TKIs: Epidermal Growth Factor Receptor Tyrosine Kinase Inhibitors
pEGFR: Plasma EGFR
pEGFRmut: Plasma EGFR Mutation
ex19del: Deletion of Exon19
cfDNA: Cell-free DNA
tEGFR: Tumor EGFR
tEGFRmut: Tumor EGFR Mutation
RECIST: Response Evaluation Criteria in Solid Tumors
PFS: Progression Free Survival
OS: Overall Survival
PR: Partial Response
SD: Stable Disease
PD: Progressive Disease
HR: Hazard Ratio
CI: Confidence Interval
ctDNA: Circulating Tumor DNA
ARMS: Amplification Refractory Mutation System
SE: Standard Error
PPV: Positive Predictive Value
NPV: Negative Predictive Value
