# Supplementary material for: Estimation of cell-free circulating EGFR mutation concentration predicts outcomes in NSCLC patients treated with EGFR-TKIs
Source: Oncotarget. 2017 Jan 4;8(8):13195–205. doi: 10.18632/oncotarget.14490 (PMC5355088; doi:10.18632/oncotarget.14490)
Supplement: Supplementary file 1 [file oncotarget-08-13195-s001.pdf]

Estimation of cell-free circulating *EGFR* mutation concentration predicts outcomes in NSCLC patients treated with EGFR-TKIs

SUPPLEMENTARY DATA

DETAILED METHOD FOR COLLECTION OF CIRCULATING CELL FREE DNA AND DDPCR WORKFLOW

Circulating cell free DNA collection

Baseline plasma was collected from each patient before first-line therapy. Additional plasma was collected at follow-up every 2 months, if possible, for patients who received EGFR-TKIs, with a maximum interval of 3 months. 6-10ml of whole venous blood were collected into EDTA-containing vacutainers, stored at 4°C before centrifuging, and centrifuged for 10 min at 1800g and 4°C within 6 hours of collection. Plasma was frozen at -80°C until use. Before DNA extraction, plasma was further cleared by centrifugation for 10 min at 3000g and 4°C. Circulating cell free DNA was isolated using the QIAmp circulating nucleic acid kit (Qiagen) according to the manufacturer's protocol. DNA was eluted in AVE buffer (100 µl) and stored at -80°C until use.

Droplet digital PCR workflow

The droplet digital polymerase chain reaction PCR (ddPCR) workflow was conducted at WuXiAppTec Medical Testing Institute(Shanghai) Co., Ltd. Scientists conducting ddPCR were blinded to the tissue results. TaqMan PCR reaction mixtures were assembled from a 2× ddPCR Mastermix (Bio-Rad Laboratories, Hercules, CA, USA) and custom-made 40x TaqMan probes/primers (Life Technologies) specific for each assay. 4µl of template DNA and 16µl of assembled ddPCR reaction mixture and pure distilled water were loaded into sample wells of an eight-channel disposable droplet generator cartridge (Bio-Rad). An additional 70µl of droplet generation oil (Bio-Rad) was loaded into the oil well for each channel. After droplet generation, the cartridge was removed and the droplets were manually transferred with a multichannel pipet to a 96-well PCR plate. The plate was heat-sealed, placed on a conventional thermal cycler, and amplified to the end-point. After PCR, the 96-well PCR plate was read on the QX-100 droplet reader (Bio-Rad). Analysis of ddPCR data was performed with QuantaSoft analysis software (Bio-Rad) that accompanied the droplet reader.

Droplet digital PCR reagents were ordered from Bio-Rad. Primer/probe mix for EGFR T790M, EGFR L858R, and EGFR exon 19 deletion were custom-made by Life Technologies. The allele-specific MGB probes were labeled with either VIC or FAM at the 5' end and a nonfluorescent quencher (NFQ) at the 3' end.

For EGFR L858R assay, primer sequences were: forward, 5'-GCAGCATGTCAAGATCACAGATT-3', reverse, 5'-CCTCCTTCTGCATGGTATTCTTTCT-3'; probe sequences were: 5'-VIC-AGTTTGGCCAGC CCAA-MGB-NFQ-3', 5'-FAM-AGTTTGGCCCGCCCAA-MGB-NFQ-3'.

For EGFR ex19del ddPCR assay, primer sequences were: forward, 5'-GTGAGAAAGTTAAA ATTCCCGTC-3', reverse, 5'-CACACAGCAAAGCAGA AAC-3'; probe sequences were: 5'-VIC-ATCGAGG ATTTCCTTGTTG-MGB-NFQ-3', 5'-FAM-AGGAATT AAGAGAAGCAACATC-MGB-NFQ-3' (ex19 deletion hotspot).

For EGFR T790M assay, primer sequences were: forward, 5'-GCCTGCTGGGCATCTG-3', reverse, 5'-TCT TTGTGTTCCCGGACATAGTC-3'; probe sequences were: 5'-VIC-ATGAGCTGCGTGATGAG-MGB-NFQ-3', 5'-FAM-ATGAGCTGCATGATGAG-MGB-NFQ-3'.

The cycling conditions for L858R and T790M were 95°C x 10 min (1 cycle), 40 cycles of 94°C x 30 s and 58°C x 1 min, followed by 10°C hold. The cycling conditions for ex19del were 95°C x 10 min (1 cycle), 40 cycles of 94°C x 30 s and 55°C x 1 min, followed by 10°C hold.

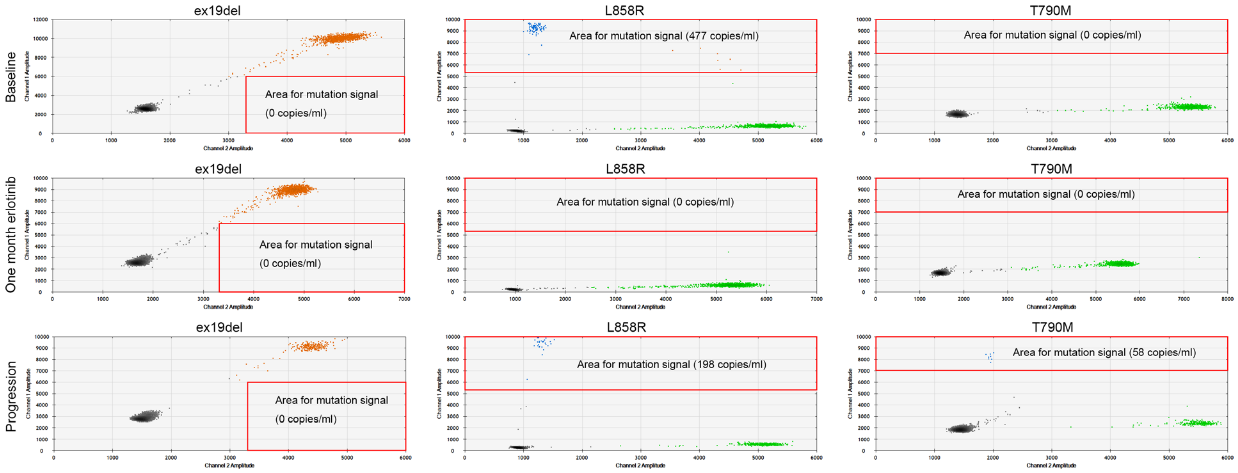

Supplementary Figure 1: ddPCR flow cytometry plots from a patient with baseline plasma L858R mutation, negative plasma *EGFR* mutation after one month of erlotinib, and positive L858R and T790M mutations at progression. Ex19del, exon 19 deletion.

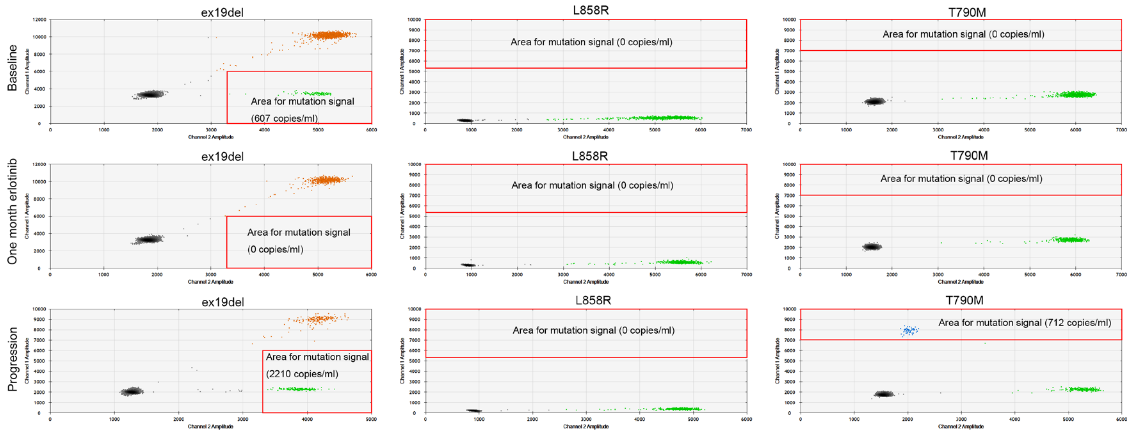

Supplementary Figure 2: ddPCRflow cytometry plots from a typical patient with baseline plasma exon19 deletion (ex19del), negative plasma *EGFR* mutation after one month of erlotinib, and positive ex19del and T790M mutations at progression.
